# Regulatory and evolutionary signatures of sex-biased genes on both the X chromosome and the autosomes

**DOI:** 10.1186/s13293-017-0156-4

**Published:** 2017-11-02

**Authors:** Jiangshan J. Shen, Ting-You Wang, Wanling Yang

**Affiliations:** Department of Paediatrics and Adolescent Medicine, LKS Faculty of Medicine, The University of Hong Kong, 21 Sassoon Road, Pokfulam, Hong Kong

**Keywords:** Sexual dimorphism, RNA-seq, X inactivation, Sex-biased gene expression

## Abstract

**Background:**

Sex is an important but understudied factor in the genetics of human diseases. Analyses using a combination of gene expression data, ENCODE data, and evolutionary data of sex-biased gene expression in human tissues can give insight into the regulatory and evolutionary forces acting on sex-biased genes.

**Methods:**

In this study, we analyzed the differentially expressed genes between males and females. On the X chromosome, we used a novel method and investigated the status of genes that escape X-chromosome inactivation (escape genes), taking into account the clonality of lymphoblastoid cell lines (LCLs). To investigate the regulation of sex-biased differentially expressed genes (sDEG), we conducted pathway and transcription factor enrichment analyses on the sDEGs, as well as analyses on the genomic distribution of sDEGs. Evolutionary analyses were also conducted on both sDEGs and escape genes.

**Results:**

Genome-wide, we characterized differential gene expression between sexes in 462 RNA-seq samples and identified 587 sex-biased genes, or 3.2% of the genes surveyed. On the X chromosome, sDEGs were distributed in evolutionary strata in a similar pattern as escape genes. We found a trend of negative correlation between the gene expression breadth and nonsynonymous over synonymous mutation (dN/dS) ratios, showing a possible pleiotropic constraint on evolution of genes. Genome-wide, nine transcription factors were found enriched in binding to the regions surrounding the transcription start sites of female-biased genes. Many pathways and protein domains were enriched in sex-biased genes, some of which hint at sex-biased physiological processes.

**Conclusions:**

These findings lend insight into the regulatory and evolutionary forces shaping sex-biased gene expression and their involvement in the physiological and pathological processes in human health and diseases.

**Electronic supplementary material:**

The online version of this article (10.1186/s13293-017-0156-4) contains supplementary material, which is available to authorized users.

## Background

Despite sex being an important epidemiological factor in disease prevalence and severity, genetic studies often do not explicitly study sex as a variable. Studies into the genes that have sex-biased gene expression, both on the autosomes and on the X chromosome, and into the regulatory and evolutionary forces that sculpt these genes to be sex biased will have implications for both evolutionary and medical genetics. In this study, we used RNA-Seq data from the Geuvadis consortium [1] to determine the sex-biased gene expression in the lymphoblastoid cell line (LCL). Evolutionarily, sex-biased gene expression may be the result of sexual or natural selection, including possibly differing selection pressures between sexes [2]. Studies of sex-biased gene expression on the X chromosome and on autosomes can help us understand the different types of selection pressures at play and the extent to which they can influence sexual dimorphism. In terms of gene regulation, many epigenetic marks may be used to study the gene regulation of sexually dimorphic gene expression. Chromatin accessibility refers to the eviction of nucleosome, allowing transcription factors to bind, and regions of chromatin accessibility are regarded as regions that contain important regulatory elements such as promoters and enhancers [3]. Promoters and enhancers are further marked by the presence of specific histone modifications, such as H3K4me3. In addition, replication timing refers to the order in which segments of DNA are replicated and have been found to be correlated with level of transcription and evolutionary conservation [4]. Recently, all of histone modification marks [5], chromatin accessibility [6, 7], and replication timing [7] have been found to have sex-specific elements, leading to questions about what we can learn about gene regulation and evolution from these genomic features. In this study, we use a variety of genomic features and evolutionary measurements to investigate regulatory and evolutionary forces on sex-biased genes.

Like all gene regulation, regulation of sex-biased gene expression is complex and dependent on a multitude of factors such as transcription factors, enhancers, super enhancers, nuclear positioning, and 3D structures of the chromatin [8]. In addition, sex-biased gene regulation is different on the X chromosome compared to the autosomes. On the X chromosome, *XIST*, a long non-coding RNA, regulates the inactivation of one of the X chromosomes in females. X inactivation governs the amount of gene expression in females and is one of the main factors determining sex-biased gene expression on the sex chromosome. Genes that escape X inactivation tend to be female biased in their gene expression but may also be male biased [9]. Despite the amount of knowledge we have about X inactivation, not all genes that escape X inactivation have been found: recent studies highlighted the variation among individuals in the genes that escape X chromosome inactivation (XCI) (escape genes) [10]. In this study, we classify all the escape genes in the 246 females using a statistical method that tests whether the allelic expression of X-linked genes are skewed outside of the range expected due to the sample clonality.

On autosomes, less is known about sex-biased gene expression. Kukurba et al. [6] detected sex-biased chromatin accessibility, defined as regions of the genome where males and females have different height of chromatin accessibility peaks. These regions correspond to genes regulated by sex-specific expression quantitative trait loci (eQTL) and were enriched in genes with sex-biased expression, implying that the accessibility and 3D structure of chromatin may play a role in sex-biased gene-expression (6). Topologically associating domains (TADs) not only delineate the 3D boundaries of transcription but also correspond to boundaries of replication timing domains [11]. On the human X chromosomes, escape genes tend to be clustered in TADs and the actively transcribed X chromosome (Xa) has an orderly replication timing whereas the inactivated X chromosome (Xi) was found to have random replication timing [7]. This corresponds well with the genome-wide observation that late replication and transcriptionally inactive regions on the autosomes are also replicated in an unstructured manner, suggesting that a strict replication timing program is involved in gene regulation [7]. In that vein, replication timing has been found to be correlated to levels of transcription and level of evolutionary conservation [4]. In this study, we investigate whether there are differences in replication timing in male- and female-biased genes, and whether specific regions of the chromosomes are enriched in sex-biased gene expression, both linearly along the chromosome and in the 3D topologically associated domains (TADs).

Evolutionarily, many forces affect the evolution of sex-biased gene expression genome-wide. Rice and Chippindale [12] have proposed that sexual antagonism is a driving force in sex-biased gene expression genome-wide, as sex-biased gene expression would maximize the fitness of both sexes. Mank et al. [13] showed that in some species, pleiotropy affects the ability of sex-biased genes to resolve the sexual antagonism and that sex-biased genes evolve more slowly due to pleiotropy. In Drosophila, male-biased genes have higher recombination rates, in addition to higher dN/dS ratio [14], a phenomenon attributed to the male genes being under positive selection and resolving sexual antagonism through higher rates of recombination [14]. As the Mank and Hultin-Rosenberg study used expression data from chickens and mice, it is not clear whether this trend holds in humans. On the X chromosome, genes have evolved in evolutionary strata, where genes of similar evolutionary age cluster in recombination blocks due to the gradual loss of recombination between these blocks and the Y chromosome [15]. The strata can be further grouped into X-added regions (XAR) and X-conserved region (XCR). Although it is known that genes that escape X inactivation are more likely to be found in younger evolutionary strata on the X chromosome [10], it is not clear whether sex-biased gene expression also follow that pattern. As well, there has been conflicting reports on whether genes that escape X inactivation (escape genes) experience different amounts of selection pressure than genes that do not [10, 16]. We also seek to answer these questions in this study.

Overall, we aimed to identify the genes with sex-biased expression on both the autosomes and the X chromosome in 462 samples and to investigate the regulation and evolutionary forces acting on such genes. On the X chromosome, we used a novel method that takes into account the clonality of the LCLs in order to demarcate genes into silent genes that are subject to X chromosome inactivation and ones that escape XCI (escape genes). On the autosomes, we used a method tailored to RNA-seq data to call differentially expressed genes between the sexes. We investigated selection pressure experienced by genes that escape XCI, as well as sex-biased genes, taking into account a variety of factors such as the level of gene expression, dN/dS, and pleiotropic constraint as measured by breadth of gene expression across tissues. Genome-wide, we investigated the markers of gene regulation such as chromatin accessibility, replication timing, 3D structure, and their role in regulating sex-biased gene expression. In addition, we performed pathway analyses and transcription factor enrichment analyses in order to gain insight into the diseases that the sex-biased genes are enriched in. In the end, these data will give us more understanding of the genes that are sex biased, how they are regulated, and the evolutionary pressure they face.

## Methods

### Identifying monoallelic expression of X-linked genes

With the overall aim of characterizing the number of genes that undergo X chromosome inactivation (XCI) and the variability of that number in our study population, we performed variant calling on RNA-Seq data to identify the genes subject to XCI. RNA-seq data from the Geuvadis consortium [1] was used, which included whole transcriptome sequencing data on 462 individuals from Northern European from Utha (CEU) and Yoruba (YRI) populations. Genotype data of the same group of individuals were downloaded from the 1000 Genomes Project Phase 3 data on November 26, 2014. We adapted the method from Lappalainen et al. [1] to determine whether a gene is expressed from one or both alleles. SAMtools was used for quality control [17], and we followed the Genome Analysis Toolkit (GATK) RNA-seq best practices on variant calling to call alternative alleles [17–19]. The quality filtering criteria were as follows:

- Read with distance to reference (NM) ≤ 6 and mapping quality (MQ) > 175 were kept.
- Sites with less than 20-fold coverage were filtered out.
- Regions with known RNA editing site based on the database DARN [20] and non-uniquely mappable sites downloaded from UCSC [13] were filtered out.

Using the criteria above and examining only genotypically heterozygous sites, we obtained 10,369 exonic SNPs in 246 females. Two hundred thirty-four genes contained enough data to be classified into escape or non-escape genes.

### Characterization of XCI

Allelic ratios have previously been used to ascertain the escape status of genes [9]. In monoclonal samples, escape genes would likely have biallelic expression whereas silent genes would have mono-allelic expression. In clonal cell lines, the silenced genes are expected to match the clonality of the cell lines while escape genes are not expected to [9]. In order to account for the skew in allelic ratio caused by polyclonality in LCL [21], we used a curated list of known silent genes [9] to estimate the allelic ratio in silent genes. For each individual, we estimated the mean and standard deviation of allelic ratio from the list of known silent genes, which gave us an estimate of allelic ratios that reflect that individual sample’s clonality. At biallelic sites, the allelic ratios were calculated as

$$ \mathrm{allelic}\  \mathrm{ratio}=\mathrm{coverage}\  \mathrm{of}\  \mathrm{the}\  \mathrm{major}\  \mathrm{allele}/\mathrm{total}\  \mathrm{coverage}\ \mathrm{at}\ \mathrm{that}\  \mathrm{site}. $$

The mean and standard deviation of allelic ratios in the known silent genes were used to form the normal distribution, against which we tested the skew in allelic ratio in the other genes. For a given individual, if the allelic ratio of a gene were significantly different than the allelic ratio calculated from silent sites, then it is designated as an escape gene in that individual. Population-wide, if gene is designated as an escape gene in more than 30% of the individuals, we classified this gene as an escape gene overall. The 30% cutoff was picked based on maximizing sensitivity and specificity in calling known silent and escape genes. Based on this 30% cutoff, the misclassification rate of known escape genes is 3% and the area under curve (AUC) is 0.92 (Additional file 1: Figure S1).

### Sex-biased gene (sDEG) characterization

We used the R package TweeDESeq [22] to compare gene expression between males and females. TweeDESeq first normalizes the raw counts of RNA-Seq reads, and then fits the RNA-Seq count data to a family of flexible distributions that can accommodate a variety of shapes of count distributions, such as tail heavy, Poisson, and negative binomial. This package takes advantage of the increased sample size to estimate two parameters of count distribution using maximum likelihood. Benjamini-Hochberg false discovery rate (FDR) adjusted *P* value of 0.05 was used as a cutoff, where genes with adjusted *P* values below the cutoff were determined as having sex-biased expression. This was performed for all samples together, and for the CEU population alone (*N* = 338, 161 males, 177 females) and the YRI population alone (*N* = 124, 55 males, 69 females).

### Transcription factor binding analysis

Information on transcription factor binding sites (TFBS) based on a combination of evidence from ChipSeq data and DNase hypersensitive sites was downloaded from ENCODE [23]. The binding sites of these transcription factors were separated into proximal transcription factors, which are sites within 2 kb of the transcription starting site (TSS) of genes, and distal TFBS, which were all other TFBS. We were interested in the enrichment of proximal TFBS in the proximal regulatory regions of sDEGs, where sDEGs were defined as genes with nominal *P* value < 0.05 in the DEG analysis. We performed a permutation test where we permuted the gene list 1000 times from a reference gene set of all ENSEMBL genes so that each time we have a randomly drawn gene list that contain the same number of genes as the input sDEG list. We counted the binding of each TF in the proximal regulatory region each time. This formed the null distribution against which we evaluated the observed TF binding counts. We performed this permutation test for both the female-biased genes, male-biased genes, and both combined. Out of the 91 ENCODE cell lines used, 38 were female, 26 male, and 27 unable to be classified as either.

### Functional and pathway enrichment analysis

Functional enrichment analyses for escape genes and for sex-biased genes were performed using the ToppFun function of the ToppGene suite [24](accessed on July 28, 2015 from https://toppgene.cchmc.org/enrichment.jsp). The 14 categories tested ranged from GO terms, disease gene sets to molecular and biochemical pathways. ToppFun also has databases of coexpression gene sets, where genes that are coexpressed are curated from MSigDB, gene expression atlas, or literature.

For genes that are differentially expressed between the sexes, an additional pathway analysis was performed using Gene Set Enrichment Analysis (GSEA) [25]. In this case, we ranked the genes by log2 fold change in gene expression between sexes and used GSEA’s “pre-ranked gene” option to look for pathways that are enriched in MSigDB. Because of GSEA’s ability to take rank into account, this analysis allowed us to detect the pathways in which the male-biased genes are upregulated separately from the pathways female-biased genes are enriched in, without having to separately evaluate each. As well, GSEA allowed for input of custom gene sets, from which we can determine whether the pre-ranked list of genes is enriched in these gene sets. We input three custom gene sets curated from the following sources involving disease genes ranging from systemic lupus erythematosus (SLE_YANG) [26], rheumatoid arthritis (RA_2104) [27] to schizophrenia [28].

### Analysis of selection pressure

Ratio between nonsynonymous and synonymous substitutions (dN/dS) can be used as a measure of selection [16]. We obtained dN/dS for macaque-human orthologs from ENSEMBL release version 83 [29]. On the X chromosome, we were interested in whether the selection pressure on escape genes differed than those on silent genes. Factors that have been shown to influence selection pressure of X-linked genes include the evolutionary strata a gene is in, whether it has a homolog on the Y chromosome and its gene expression level. We therefore tested the difference in selection pressure between escape and silent genes first using univariate regression, and then using the multiple regression model allowing for covariates:

$$ y\sim \mathrm{b}0+\mathrm{b}1x1+\mathrm{b}2x2+\mathrm{b}3x3+\mathrm{b}4x4+\mathrm{b}5x5+e $$

where *y* is dN/dS for a X-linked gene. *x*1 is a discrete variable that denotes whether the gene is part of the X added region (XAR) vs X conserved region (XCR). *x*2 is a discrete variable indicating whether the gene is part of an XY pair, *x*3 is a continuous variable denoting the average gene expression of the X-linked gene, *x*4 denotes the escape status in two levels (escape gene or non-escape gene), *x*5 denotes whether it was classified as a disease gene in OMIM genes [30], and *e* denotes the error term.

On the autosome, we were interested in the amount of selection pressure experienced by sex-biased genes. The breadth of gene expression is one measure of pleiotropy and a factor that have been shown to affect the amount of selection experienced by a gene [13]. Gene expression breadth in this study is measured by the number of tissues a gene is expressed in. We downloaded the FANTOM5 consortium data from Gene Expression Atlas on September 30, 2015 and tallied up the number of tissues a gene is expressed in to obtain a gene expression breadth value ranged between 1 and 56. Out of the 1309 samples used in FANTOM5, 304 were female, 429 male, 208 mixed, and 449 are unknown. We used univariate regression to test the difference in selection pressure and gene expression breadth between the sex-biased gene groups (female biased, male biased, and non-biased). We then investigated whether gene breadth varied between male- and female-biased genes while adjusting for covariates such as dN/dS using the linear regression with multiple covariates:

$$ y\sim \mathrm{b}0+\mathrm{b}1x1+\mathrm{b}2x2+\mathrm{b}3x3+e $$

where *y* represents gene breadth, *x*1 is the discrete variable that denotes sex bias (female biased, male biased, and non-biased), *x*2 is the continuous variable dN/dS between macaque and humans, *x*3 gene expression averaged across all samples, and *e* is the error term. Furthermore, the correlation between sex bias of gene expression on dN/dS or gene expression breadth were tested with univariate regression

$$ y\sim \mathrm{b}0+\mathrm{b}1x1 $$

where *y* represents gene expression breadth, or dN/dS and *x*1 is the discrete variable that denotes sex bias (female biased, male biased, and non-biased). This was tested both in the LCL data and the Genotype-Tissue Expression Project (GTex) consortium data.

We further analyzed selection pressure on androgen-regulated and estrogen-regulated genes. Androgen-regulated genes were obtained from [31] while estrogen-regulated genes were downloaded from ESR1 from ENCODE experiment (ENCFF029ZUJ).

### Genomic distribution of sDEG and replication timing

Genomic distribution of sDEGs were investigated in two ways: through using GSEA on positional gene sets (C1 in MSigDB) and through analysis of topologically associated domains (TADs). GSEA analysis was performed as documented above. TADs were downloaded from [11]. Entropy was calculated for TADs that contained sex-biased genes. Entropy per TAD was calculated by –pi × log(pi), where pi = proportion of sex-biased gene in TAD i.

In order to investigate the relationship between replication timing and sex bias of genes, replication timing for LCL was downloaded from Koren et al. [7] and cell type-specific replication timing (wavelet-smoothed signals) was downloaded from ENCODE [23] for cell types IMR-90 (female), SK-N-SH (female), and NHEK (sex undefined). In all cases, lower values denote later replication. We calculated the average replication timing per gene as:

$$ \mathrm{Replication}\  \mathrm{timing}\ \mathrm{per}\ \mathrm{gene}\kern0.5em =\kern0.5em \frac{\sum_iR\times \mathrm{bpOverlap}}{\mathrm{totalBpInGene}} $$

Where R refers to the replication timing value, bpOverlap refers to the overlapped basepairs between replication timing domains and gene, and totalBpInGene refer to the total number of basepairs in the gene. For cell lines IMR-90, SKN-N-SH, and NHEK, replication timing is measured across each cell line, each of which is composed of a single sample. Replication values vary between 0 and 1 and are normalized from sequence read number per cell cycle, with bigger replication values representing earlier replication. The exception is LCL, where replication timing data is gathered from six samples (two females, three males, one unknown). The replication timing value is averaged per gene across all samples and the replication values vary between − 1 and 1, with bigger replication timing values representing earlier replication.

We analyzed the correlation between replication timing and sex bias of gene expression in several ways. We first performed a univariate regression between per gene replication timing values in three ENCODE cell lines IMR-90, SKN-N-SH, and NHEK and the log2 fold change in gene expression between females and males in the GTex consortium. In addition, we also used univariate regression to test the difference in the mean per gene replication timing value between female biased, male biased, and unbiased using replication data from [7] and gene expression data from the Geuvadis consortium, as they are both based on the LCL.

## Results

### Differentially expressed genes (sDEGs) between sexes

Using the package TweeDESeq and a Benjamini-Hochberg FDR cutoff of 0.05, we identified 587 genes genome-wide that are differentially expressed between males and females in the LCLs, which accounted for 3.2% of the genes surveyed. In total, the numbers of male- and female-biased genes found are similar: 318 genes are found to be female biased and 269 genes male biased. On the X chromosome, however, there are more female-biased genes: 64 X-linked genes are female biased and 20 X-linked genes are male biased.

When sDEGs are analyzed between populations, some genes are sex biased in both populations, while some are sex biased in only one population. Sixty-eight genes are found to be sex biased in YRI, while 510 are found to be sex biased in CEU population. In YRI, 47 genes are female biased whereas 21 genes are male biased. In the CEU population, 300 genes are female biased whereas 210 genes are male biased. Few genes were found to be sex biased in only one population: 5 genes in YRI and 172 genes in CEU were population-specific sDEGs. The concordance of sDEGs between the two populations is 65.5%, showing that most sDEGs are common across the two populations. When the log2fc between female- and male-biased gene expression, or effect sizes, of the gene expression bias are compared between the populations, there is a strong correlation (Pearson’s correlation = 0.71, *P* value < 2.1e−16).

### Genes that escape XCI are differentially distributed across evolutionary strata on the X chromosome

**Fig. 1:**
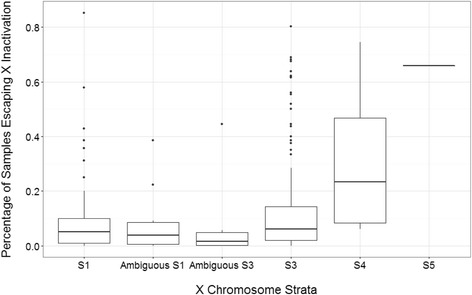
Percentage of samples that escape X inactivation across different evolutionary strata. Each data point plots the number of samples that express both copies of a gene. Strata are organized by evolutionary time and position on the X chromosome, where S1 is the oldest stratum and S5 the youngest stratum. All genes are supported by at least five data points in the population

We used GATK heterozygous calls on expression data to determine biallelic expression of X-linked genes in 246 females. We found 35 genes escaping XCI out of 286 genes surveyed. Two hundred fifty-one genes are found to be lacking of evidence of escaping XCI, including the 110 previously listed silent genes. In accordance with previous studies, a significantly higher percentage of genes escaping XCI are found in younger evolutionary strata (univariate regression, *P* value = 0.00253) (Fig. 1).

### Female-biased gene expression and X chromosome

**Fig. 2:**
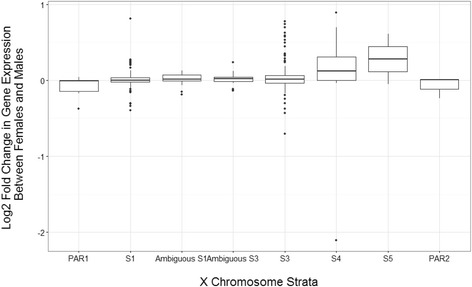
Change in gene expression between female and male samples in across X chromosome strata. Positive log fold changes are classified as female-biased genes, whereas negative log fold change genes were classified as male-biased genes

If sex-biased expression on the X chromosome were due to genes escaping XCI, then we would expect a significant overlap between genes that have an expression bias and genes that escape XCI. We tested this hypothesis and found a correlation between the log2 fold change in gene expression between female and male samples and percentage of samples in the population that escape XCI for that gene (Pearson’s *R* = 0.788, *P* < 2.2e−16). There is a slight trend that younger strata are more likely to have genes that are female biased (univariate regression, *P* value = 0.024) (Fig. 2).

### Evolutionary pressure on escape genes and sex-biased genes

**Fig. 3:**
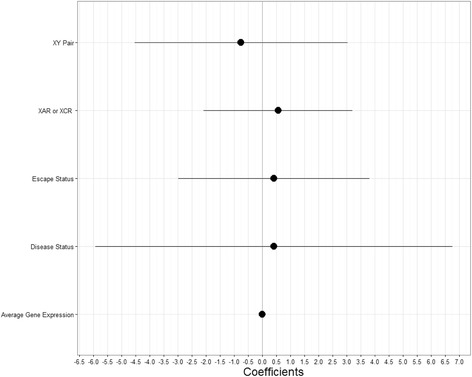
Selection pressure dN/dS on escape genes as a measure of the covariates. Coefficients for linear regression of dN/dS, or the amount of selection pressure, as a function of the following variables. XY pair refers to whether the gene is part of an XY homologous pair, where the coefficient is the genes that are part of XY pairs compared to genes that are not. XAR or XCR refers to whether the gene belongs on the X added region on the X chromosome or the X conserved region strata, where the coefficient is for XCR compared to XAR. Escape status refers to whether the gene is classified as an escape gene, where the coefficient is for escape genes compared to non-escape genes. Average gene expression refers to the average level of gene expression among 264 female samples. Disease status refers to whether the gene was classified as a disease gene in OMIM

We measured the evolutionary pressure undergone by escape genes by comparing the difference in mean macaque-human dN/dS between escape and silent genes. We first performed univariate regression using the single variable of escape status (classified as escaper or non-escape genes), and then used a multiple regression model that accounts for a variety of covariates. No relationship was found between dN/dS and escape status of a gene (univariate regression *P* value = 0.46). This result held when we expanded our multiple regression model using as covariates whether the gene was part of an XY pair, whether it was classified as a disease gene from OMIM, whether it was in the X-added region (XAR) or X-conserved region (XCR) and the average gene expression level (Fig. 3, Additional file 2: Table S1). This is true whether we use the set of genes that we have classified as escape (multiple regression, *P* value = 0.92) or the set of genes that others have classified escape [9] (multiple regression, *P* value = 0.74).

**Fig. 4:**
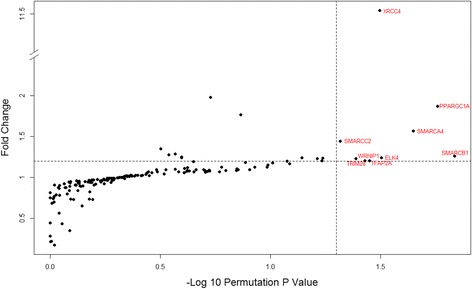
Change in the nonsynonymous mutation over synonymous mutation ratio against gene expression breadth. We show data from both European (CEU) and Yoruba (YRI) populations surveyed, separated by sex. Blue line denotes the slope of the line under a linear model, while the gray shade denotes the 95% confidence interval from the univariate regression model. Beta and *P* value denotes beta coefficient between gene expression breadth and dN/dS in the univariate analysis, and the corresponding *P* value

Sexual antagonism may be a driving force behind sex-biased gene expression, and pleiotropy may constraint the sex-biased gene’s ability to evolve in a way that benefits both sexes [2]. In this study, we tested the amount of selection pressure that sex-biased genes are under (estimated by dN/dS) and its relationship with gene expression breadth (as a measure of pleiotropy). In our study, dN/dS is not significantly different between male biased, female biased, or non-biased genes in LCL as a whole (Table 1). This lack of difference in dN/dS is also seen when we analyze genes that are sex biased in tissues in the GTex consortium (Table 2), or when we analyze the CEU and YRI population together or separately (Tables 3, 4, and 5). On the other hand, gene expression breadth is negatively correlated with dN/dS in LCL overall (Table 6), whether we account for gene expression level and sex bias in gene expression or not (Additional file 2: Table S2), and whether we use sex-biased genes found from tissues in the GTex consortium or LCL (Tables 2, 3, 4, and 5). The trend also remains when we analyze the two CEU and YRI separately (Fig. 4). Overall, the general trend is a lower, but not statistically significant, dN/dS in males as compared to unbiased genes and simultaneously a lower gene expression breadth in male-biased genes as compared to unbiased genes (Tables 3, 4, and 5). When we compared genes regulated by androgen to those regulated by estrogen, we found androgen-regulated genes had higher dN/dS than estrogen-regulated genes (univariate regression, *P* value = 0.0325).

**Table 1:** Mean nonsynonymous over synonymous rate (dN/dS), gene expression breadth, and their correlations in male, female, and unbiased genes for LCL samples including both populations

	Male	Female	Unbiased	*P* ANOVA between all 3	*P* ANOVA between sex-biased and unbiased	*P* ANOVA between male and female
dN/dS	0.2332364	0.2773688	0.2813096	0.815	0.634	0.107
Gene expression breadth	44.72727	43.57561	45.89424	0.0637	0.0259	0.481
Correlation between dN/dS and gene expression breadth	Spearman’s rho = 0.03181803; *P* value = 0.6655	Spearman’s rho = − 0.1276786; *P* value = 0.0681	Spearman’s rho = − 0.01899588; *P* value = 0.0499	(All data together) Spearman’s rho = − 0.01938879; *P* value = 0.0415

**Table 2:** Mean nonsynonymous over synonymous rate (dN/dS), gene expression breadth, and their correlations in male and female sex-biased genes from the GTex consortium. Beta denotes coefficients in the univariate model

	Male bias	Female bias	*P* univariate regression between male and female
dN/dS	0.2648424	0.3001547	0.0133
Gene expression breadth	32.26667	27.70866	0.661
Correlation between dN/dS and gene expression breadth	Univariate regression beta = − 0.003392; *P* value = 0.0854	Univariate regression beta = − 0.000999; *P* value = 0.362

**Table 3:** Mean nonsynonymous over synonymous rate (dN/dS), gene expression breadth, and how they vary according to sex bias of gene expression in LCLs containing both CEU and YRI population samples

	dN/dS beta	dN/dS *P* value	Gene breadth beta	Gene breadth *P* value
Intercept	0.2813	< 2e−16*	45.89	< 2e−16*
Male bias compared to unbiased	− 0.048	0.524	− 1.1670	0.3055
Female bias compared to unbiased	− 0.00394	0.956	− 2.3186	0.0332

All analyses were carried out using a univariate regression model with sex as a covariate. Betas denote coefficients in the univariate regression model, while *P* value denotes the *P* value of the coefficient

*Statistical significance at *P* value < 0.05 cutoff

**Table 4:** Mean nonsynonymous over synonymous rate (dN/dS), gene expression breadth, and how they vary according to sex bias of gene expression in CEU samples only

	dNdS beta	dNdS *P* value	Gene breadth beta	Gene breadth *P* value
Intercept	0.2819	< 2e−16*	45.8583	< 2e−16*
Male bias compared to unbiased	− 0.087579	0.275	− 0.158	0.874
Female bias compared to unbiased	− 0.011096	0.883	− 0.9381	0.409

All analyses were carried out using a univariate regression model with sex as a covariate. Betas denote coefficients in the univariate regression model, while *P* value denotes the *P* value of the coefficient

*Statistical significance at *P* value < 0.05 cutoff

**Table 5:** Mean nonsynonymous over synonymous rate (dN/dS), gene expression breadth, and how they vary according to sex bias of gene expression in Yoruba samples only

	dN/dS beta	dN/dS *P* value	Gene breadth beta	Gene breadth *P* value
Intercept	0.280676	< 2e−16*	45.8485	< 2e−16*
Male bias compared to unbiased	− 0.020105	0.962	− 19.6818	0.00179*
Female bias compared to unbiased	− 0.104992	0.608	0.7515	0.80780

All analyses were carried out using a univariate regression model with sex as a covariate. Betas denote coefficients in the univariate regression model, while *P* value denotes the *P* value of the coefficient

*Statistical significance at *P* value < 0.05 cutoff

**Table 6:** Mean nonsynonymous over synonymous rate (dN/dS) correlation with gene expression breadth, tested using univariate regression in LCL, both European and Yoruba population

	Beta	Std. Error	*P* value
Intercept	0.339	0.030	< 2e−16*
Gene expression breadth	− 0.0012821	0.0006289	0.0415

### Regulation of sex-biased genes: transcription factor binding site enrichment and pathway analysis

**Fig. 5:**
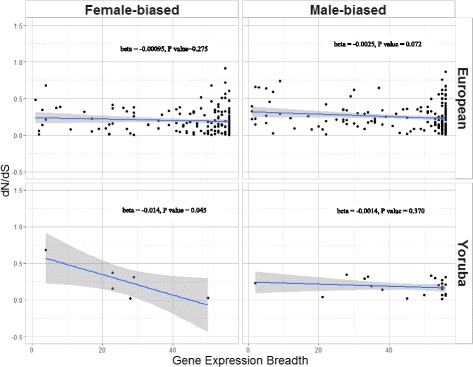
Transcription factor binding site enrichment in female-biased genes. The dotted line indicates the 0.05 permutation *P* value cutoff. Fold change refers to the number of times a TFBS is enriched in permutations relative to the observed value

No transcription factor (TF) was found to be significantly enriched in male-biased genes. Using the data on transcription factor binding sites, nine TFs were found enriched in the proximal regulatory regions of female-biased genes: SMARCB1, PPARGC1A, SMARCA4, ELK4, XRCC4, TFAP2A, TRIM28, WRNIP1, and SMARCC2. (permutation *P* value < 0.05, Fig. 5). Pathway analyses on these transcription factors revealed a variety of pathways where two or more TFs listed above are involved. These include glucocorticoid receptor regulatory network and Wnt signaling (Additional file 2: Table S9). Interestingly, glucocorticoid receptor regulatory network is known to be a sexually dimorphic pathway in human liver [32].

### Genomic distribution and replication timing

**Fig. 6:**
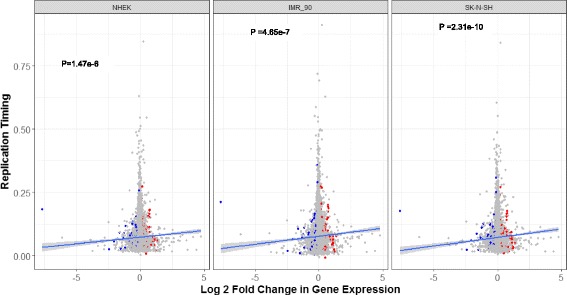
Replication timing in three ENCODE cell lines as a function of sex bias in gene expression. Higher log2 fold change values indicate female-biased gene expression, while higher replication timing values indicate earlier replication timing. Red and blue points indicate statistically significant female-biased and male-biased genes, respectively, based on genes classified as sex biased by the GTex consortium. Blue lines indicate the fitted univariate regression lines for each cell type; *P* values indicate the statistical significance of the slope

Across a variety of cell lines and tissues, female-biased genes were also more likely to be found in earlier replication timing regions. Using replication timing from LCL [7] and sex-biased gene classified using gene expression in the Geuvadis study, we find female-biased genes were more likely to be found in earlier replication timing regions as compared to male-biased genes (univariate regression, *P* value = 1.5e−12) or unbiased genes (univariate regression, *P* value = 1.81e−8) (Additional file 1: Figure S2). This trend held regardless of whether dN/dS and gene expression level were taking into account as covariates in a multiple regression model (Additional file 2: Table S4). Additionally, this trend also held when using genes classified as sex-biased from GTex consortium and compared to the replication timing values found in three ENCODE cell lines IMR-90, SK-N-SH, and NHEK (Fig. 6, Additional file 2: Table S3).

Genome-wide, there is little eVdence of sex-biased genes clustering in autosomes, although some clustering is observed on the X chromosome. Using the pathway enrichment tool GSEA, we found female-biased genes to be clustered in X chromosome as expected, but also unexpectedly in chr19q13. Using the same analysis, we also found male-biased genes to be clustered as expected on the Y chromosomes and unexpectedly on chr4q12 (Additional file 2: Table S4(a-b)). TAD analysis return similar results: most TADs do not show a greater than expected clustering of sex-biased genes (Additional file 1: Figure S3).

### Pathway enrichment

Similar to escape genes, sDEGs overall were over represented in a variety of disease and functional pathways. The program ToppFun found B cell lymphoma and chronic obstructive airway diseases were enriched in sDEGs (Additional file 2: Table S5(a)). A variety of gene families were also enriched in sDEGs (Additional file 2: Table S5(b)). None of the custom disease gene sets were enriched in sex-biased genes (Additional file 2: Table S6).

When examining female-biased sDEGs, KEGG oocyte meiosis pathway was found to be over represented in female-biased genes as expected. Interestingly, a variety of KEGG metabolic pathways were enriched, including metabolism of xenobiotics by cytochrome p450 (Additional file 2: Table S5 (b)). In male-biased sDEGs, a number of gene families related to immune-related functions were found to be enriched (Additional file 2: Table S7(a)). Enriched pathways were also found for disease genes, including genes involved in AML and head and neck cancer (Additional file 2: Table S7(b)).

## Discussion

Sex bias of gene expression is expected to reflect both the evolutionary history of genes and their physiological roles. Our study looked for pathways that sex-biased genes are enriched in, investigated patterns of gene regulation, and revealed genomic features that vary with sex-biased genes. In addition, we characterized the escape status of X-linked genes taking into account LCL multiclonality, using a new method that utilized RNA-Seq data.

### Evolutionary insights

On the X chromosome, we found that the pattern of escape tends to vary by strata, with the younger strata harboring more escape genes, a direction that is consistent with Ohno’s hypothesis and known literature. This is similar to results from previous studies [9, 10, 33] and acted as a proof of concept for our classification and analysis methods. However, unlike some previous studies that suggest escape genes are under more purifying selection [16], the escape and silent genes do not differ in dN/dS regardless of whether we take into account gene expression level, breadth of expression, whether they are classified as disease genes by OMIM, and regardless of whether we use genes that we characterized as escape or the set of escape genes from literature. A previous study also using LCL RNA-Seq data found a similar result [10]. This shows that despite the possibly different evolutionary pressures faced by escape and non-escape genes, no difference in dN/dS can be detected; indicating escape status of the gene is not the key driver of evolution on the X chromosome.

Many forces affect the evolution of sex-biased gene expression genome-wide, including on the X chromosome. Rice and Chippindale [12] have proposed that sexual antagonism is a driving force in sex-biased gene expression. However, Mank et al. [13] showed that in some vertebrate species, pleiotropy affects the ability of sex-biased genes to resolve the sexual antagonism and that sex-biased genes may be constrained to evolve at the level permitted by the tissue specificity of their gene expression. In all of mice, chicken [13], and Drosophila [34], male-biased genes were found to have higher rate of evolution and higher tissue specificity. We used gene expression breadth as a measure of pleiotropic constraint and found that as in other organisms studied, genes evolved faster when there is narrower expression breadth as shown by the negative correlation between dN/dS and gene expression breadth in both cell line and tissue data, across the two populations analyzed, and when taking other factors such gene expression level into account (Tables 1 and 2, Fig. 5, Additional file 2: Table S2). This suggests that pleiotropy does indeed constrain the evolutionary rate of genes in humans, regardless of the sex bias of gene expression, which, to our knowledge, had not been demonstrated in humans before. However, unlike the case in chicken and mouse [13] and Drosophila [34], we did not find significantly higher rate of evolution, as measured by dN/dS, in male biased as compared female biased or unbiased genes. In fact, the general trend is a lower dN/dS in males as compared unbiased genes and simultaneously a lower gene expression breadth in male-biased genes as compared to unbiased genes (Tables 3, 4 and 5). It is possible that male-biased genes in reproductive tissues may have a different pattern, as many of the fast evolving genes in Drosophila are sperm-related genes and those may not be detected as male biased in our dataset. In another human study, Gershoni and Pietrokovski [35] used 53 tissues from 533 adults in the GTex consortium to analyze the selection pressure on sex-biased genes. They found that the greater the number of tissues a gene is sex-biased in, the greater the rate of deleterious nonsynonymous mutation, suggesting that very sex-biased genes are evolving under relaxed selection pressure and experience a lack of constraint. This is different than our finding of genes experiencing constraint from pleiotropy regardless of their sex bias in gene expression. Their study was conducted in many tissue samples, allowing them to quantify the degree to which a gene is sex-biased across all tissues. They also used a different measure of natural selection: the number of deleterious nonsynonymous mutations over the number of synonymous mutation (dN/dS) which may better measure the selection pressure against deleterious mutations. Overall, although the Gershoni and Pietrokovski [35] study did not look at selective constraint from being expressed in multiple tissues, but they did reach the interesting conclusion that extremely sex-biased genes may be under less selective constraint than unbiased genes, showing that our finding of pleiotropic constraint regardless of sex bias may need further study in human tissues under different physiological conditions.

### Sex-biased genes

We found more genes with female-biased expression than male-biased expression on the X chromosome, similar to other studies [36]. The presence of male-biased expression on the X chromosomes in LCL was unexpected, as many of the previously characterized male-biased genes on the X chromosome were testis specific. Functionally, the X-linked male-biased genes in LCL were found to be related to non-reproductive functions, such as height and cell adhesion. They were located in strata PAR1 and 2, S1, and S3, S4, with the majority of PAR1 genes being male biased, similar to the finding of Tukiainen et al. [37].

The majority of sDEGs found were not population specific, and there was high correlation in the effect size of sDEG between the two populations. More sDEGs were CEU specific than YRI specific, showing that bigger sample sizes may have led to more population-specific sDEGs detected. Alternatively, the smaller sample size in YRI may be the reason that some of the genes are only detected as sex-specific expression in CEU, and more studies with higher power are needed to address this issue in future.

### Gene regulation and link to disease

GSEA and Toppfun pathway analyses confirmed many known sex-biased pathways. “KEGG oocyte meiosis” is a known female-biased pathway that was found to be enriched for female-biased genes in our dataset. “KEGG metabolism of xenobiotics by CYP450” is a known sex-dependent pathway in rats; in humans, some studies suggest different metabolic activity of xenobiotics between males and females with genes in the CYP450 family [38, 39].

Transcription factors binding enrichment analysis can be used to assess the transcription factors that may be regulating the gene expression of sexually dimorphic genes. Eight transcription factors were found enriched in female-biased genes in our analyses although this may not uphold after multiple testing correction. Interestingly, some of the transcription factors are enriched in “glucocorticoid receptor regulatory network,” a growth hormone-mediated pathway that is known to be sexually dimorphic in rats [32]. This pathway is thought to play a role in inflammatory disease with difference in sex prevalence [32]. In humans, VGLL3 is a putative transcription factor that has recently been found to regulate female-biased inflammatory processes [36], possibly also playing a role in autoimmune diseases with a difference in sex prevalence.

We produced a novel finding of association between replication timing and sex bias of gene expression. It is not clear why female-biased genes are found in earlier replication timing regions and male-biased genes in later replicating regions. Because the replication timing domains are responsive to 3D chromatin changes and the rearrangement of genes in the cell nucleus, there is the possibility that female- and male-biased genes are repositioned spatially in response to different regulation patterns. In fact, in mice liver, sex-biased gene expression from GH signaling is maintained via sex-dependent STAT5 binding, correlating with differential chromatin accessibility between the sexes and sex-biased histone modification marks [40].

### Limitations and future directions

Evolutionarily, many factors affect the evolution of males and females differently. Mutation rate, effective population size, recombination rate [41], and pleiotropy [13, 41] all have been found to be different in male-biased, female-biased, and unbiased genes. Although we recapitulated the result that pleiotropic constraint seems to have a greater effect than sexual antagonism on the evolution of sex-biased genes, our study is limited to LCLs. As more expression data on a greater variety of tissues become available, we can better investigate how pleiotropic constraint and other factors such as recombination resolve sexual antagonism to lead to the fitness landscapes in human males and females today.

Recent advances in genomics have led to some new understanding in the regulation of sex-biased genes, such as differential chromatin accessibility in male and females [6], expression quantitative trait loci that are sex specific [6, 42] and differential gene expression in a variety of tissues [35]. However, the picture is far from complete: it is not clear how and which transcription factors interact with chromatin modification to impact gene expression to result in differential gene expression between males and females, and how replication timing or other chromosome domains may be involved. With the advances in genomic technology, we may be inching closer than ever to resolving the regulatory networks in males and females in a variety of physiological tissues and conditions.

## Conclusions

Sex-biased genes (sDEGs) are widespread phenomena across many mammalian species. We show several evolutionary trends affect sDEGs, including more sDEGs in evolutionarily later strata on the X chromosome and pleiotropic constraint on their evolutionary rate. We also discover some candidate TFs that may modify female-biased sDEGs only. The amount of functional and other data sources that are now available makes it possible to look at the features of gene regulation of sex-biased genes, the pathways they are associated with, and the forces that help shaping their evolution.

## Additional files

Additional file 1: Figure S1. Receiving operator curve (ROC) for using allele ratio of X-linked genes to predict status of escape of genes in female samples, on the X chromosome. In this figure, positive means correctly classifying a known silent gene as silent gene. The AUC for using allele ratio is 0.92. We used a cutoff of 0.3 to delineate genes between escape and silent genes, as this is the lowest allele ratio at which we have perfect classification of known silent genes. **Figure S2.** Female, male and gender non-biased genes show difference in replication timing in all the cell lines examined (ANOVA, *P* value = 0.101). Female biased genes show slightly earlier replication timing while male biased genes how later replication timing. Y axis denote replication timing values from Koren et al. (2012). **Figure S3.** Genome-wide distribution of entropy for TADs that contain sex biased genes. Lower entropy signifies better clustering of sex biased genes with other sex biased genes. **Figure S4.** Effect size in log2fc of gene expression differences of the differential gene expression analyzed separately in Utah residents with Northern and Western European Ancestry (CEU) and Yoruba (YRI) populations. For clarity, only the genes that are significantly differentially expressed by sex (sDEG) are displayed. Genes that are found to be significantly differentially expressed when both CEU and YRI populations are analyzed together are plotted in orange, while genes that are found to be sDEGs when YRI samples are analyzed alone are plotted in blue, and sDEGs in CEU samples are plotted in green. There are strong correlations in effect sizes between the two populations(Pearson’s correlation = 0.71,*p* value < 2.1e-16), and most sDEGs (65%) are shared across the two populations. (DOCX 47 kb)

Additional file 2: Table S1. Linear regression of dN/dS, or the amount of selection pressure, as a function of the following variables. Estimate refers to the coefficient of the covariate, Std.Error referes to the standard error of that estimate. T value refers to the test statistic of the estimate and Pr(>|t|) refer to the *P* value of that covariate. XYpair refers to whether the gene is part of an XY homologous pair. XAR or XCR refers to whether the gene belongs on the X added region on the X chromosome or the X conserved region strata, escape status refers to whether the gene is classified as an escape gene, and gene expression refers to the average level of gene expression among 264 female samples. **Table S2.** Linear regression of dN/dS, or the amount of selection pressure, as a function of the following variables. Estimate refers to the coefficient of the covariate, Std.Error referes to the standard error of that estimate. T value refers to the test statistic of the estimate and Pr(>|t|) refer to the p value of that covariate. Average gene expression refers to average level of gene expression among 462 samples. Gene bias_female refers to whether the gene is classified as female-biased, and the coefficient refers to the change in dnds between female-biased genes to genes without sex bias. Gene bias_male refers similarly to genes classified as male-biased. Gene expression breadth refers to the number of tissues the gene is expressed in. **Table S3.** Sex-biased genes (sDEG) (587 genes) from LCL data and GTex data (1308) and their relationship to replication timing data in different cell lines, based on the Spearman’s Rho between replication timing values and log2fc of gene expression between females and males. **Table S4(a).** GSEA results for gene regions that are enriched in female biased sDEGs. **Table S4(b).** ToppFun results for gene regions that are enriched in male-biased sDEGs. **Table S5(a).** Disease gene sets enriched in sex biased genes, as found by ToppFun. **Table S5(b).** GO terms enriched in sex biased genes, as found by ToppFun. **Table S5 (c).** Pubmed gene sets enriched in sex biased genes, as found by ToppFun. **Table S5 (d).** Pathway analysis of sex biased genes using gene sets from MSigDBC2, as found by ToppFun. **Table S5 (e).** Gene families enriched in sex biased genes, as found by ToppFun. **Table S6 (a).** Domains enriched in female biased genes, as found by ToppFun. **Table S6 (b).** KEGG pathways enriched in female biased genes, as found by GSEA. **Table S7.** Pathway analysis of sex biased genes in GSEA, using custom gene sets from [26–28, 43]. **Table S8 (a).** Gene families enriched in male biased genes. **Table S8 (b).** MSigDB gene sets enriched in male biased genes, as found by ToppFUn. **Table S9.** Pathways enriched in transcription factors enriched for female biased genes. (DOCX 49 kb)

## Abbreviations

AML: Acute myeloid leukemia
ANOVA: Analysis of variance
AUC: Area under the curve
dN/dS: Nonsynonymous over synonymous rate
eQTL: Expression quantitative trail loci
FANTOM: Functional Annotation of the Mammalian genome
FDR: False discovery rate
FWER: Family-wise error rate
GATK: Genome Analysis Toolkit
GSEA: Gene Set Enrichment Analysis
GTex: Genotype-Tissue Expression Project
IMR90: Fetal lung fibroblast
LCL: Lymphoblastoid cell line
MCF: Mammary gland epithelial
sDEG: Sex-biased differentially expressed gene
SK-N-SH: Neuroblastoma cell line
TAD: Topologically associating domain
TF: Transcription factor
TFBS: Transcription factor binding site
TSS: Transcription start site
Xa: Activated X chromosome
XAR: X-added region
XCI: X chromosome inactivation
XCR: X-conserved region
Xi: Inactivated X chromosome
