# Supplementary material for: Regulatory and evolutionary signatures of sex-biased genes on both the X chromosome and the autosomes
Source: Biol Sex Differ. 2017 Nov 2;8:35. doi: 10.1186/s13293-017-0156-4 (PMC5668987; doi:10.1186/s13293-017-0156-4)
Supplement: Supplementary file 1 — Receiving operator curve (ROC) for using allele ratio of X-linked genes to predict status of escape of genes in female samples, on the X chromosome. In this figure, positive means correctly classifying a known silent gene as silent gene. The AUC for using allele ratio is 0.92. We used a cutoff of 0.3 to delineate genes between escape and silent genes, as this is the lowest allele ratio at which we have perfect classification of known silent genes. Figure S2. Female, male and gender non-biased genes show difference in replication timing in all the cell lines examined (ANOVA, P value = 0.101). Female biased genes show slightly earlier replication timing while male biased genes how later replication timing. Y axis denote replication timing values from Koren et al. (2012). Figure S3. Genome-wide distribution of entropy for TADs that contain sex biased genes. Lower entropy signifies better clustering of sex biased genes with other sex biased genes. Figure S4. Effect size in log2fc of gene expression differences of the differential gene expression analyzed separately in Utah residents with Northern and Western European Ancestry (CEU) and Yoruba (YRI) populations. For clarity, only the genes that are significantly differentially expressed by sex (sDEG) are displayed. Genes that are found to be significantly differentially expressed when both CEU and YRI populations are analyzed together are plotted in orange, while genes that are found to be sDEGs when YRI samples are analyzed alone are plotted in blue, and sDEGs in CEU samples are plotted in green. There are strong correlations in effect sizes between the two populations(Pearson’s correlation = 0.71,p value < 2.1e-16), and most sDEGs (65%) are shared across the two populations. (DOCX 47 kb) [file 13293_2017_156_MOESM1_ESM.docx]

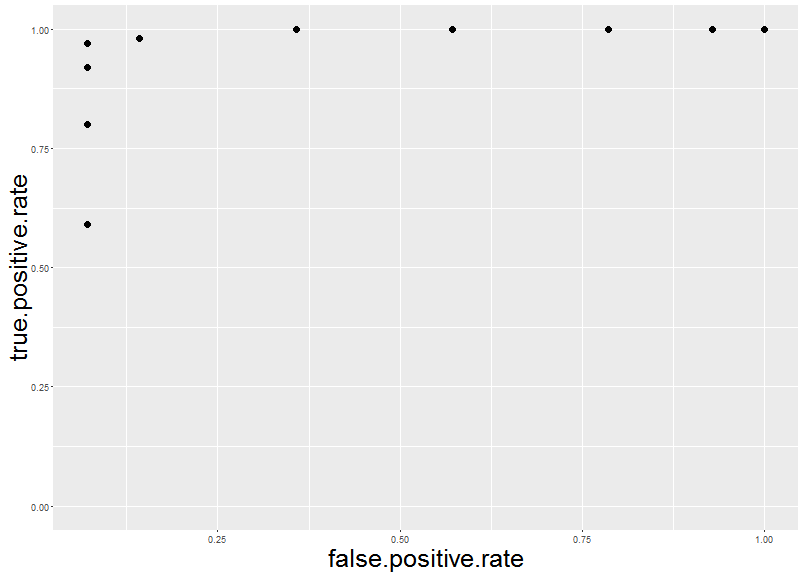


Additional file Figure 1: Receiving operator curve (ROC) for using allele ratio of X-linked genes to predict status of escape of genes in female samples, on the X chromosome. In this figure, positive means correctly classifying a known silent gene as silent gene. The AUC for using allele ratio is 0.92. We used a cutoff of 0.3 to delineate genes between escape and silent genes, as this is the lowest allele ratio at which we have perfect classification of known silent genes.


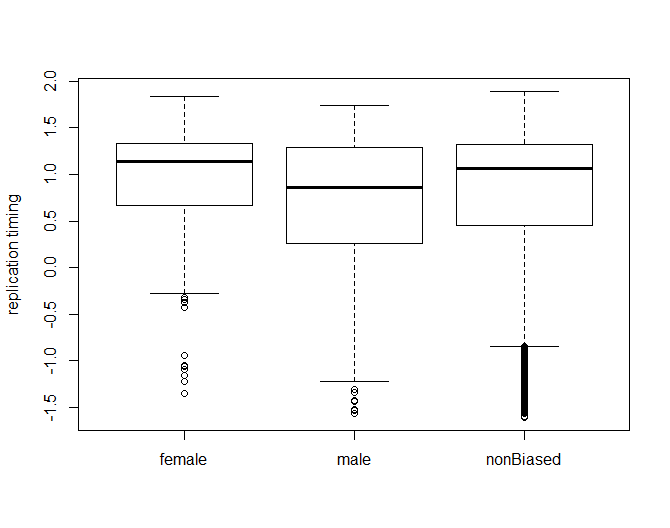


Additional file Figure 2: Female, male and gender non-biased genes show difference in replication timing in all the cell lines examined (ANOVA, p value= 0.101). Female biased genes show slightly earlier replication timing while male biased genes how later replication timing. Y axis denote replication timing values from Koren et al (2012).


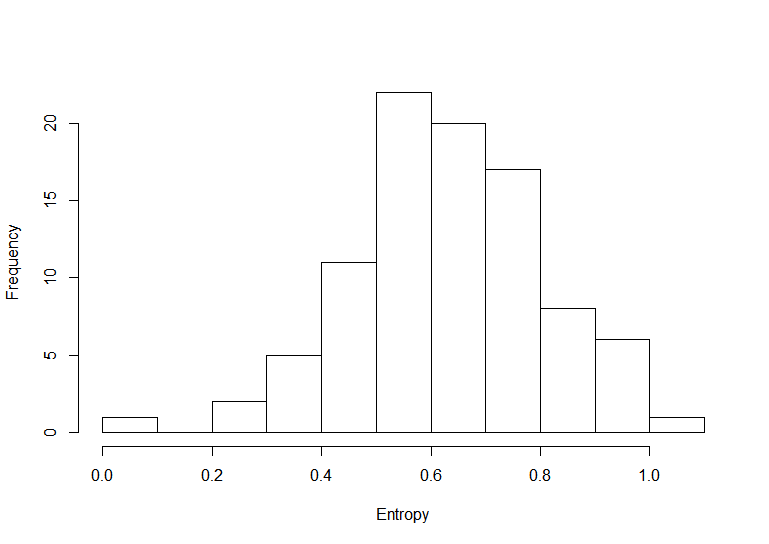


Additional file Figure 3: Genome-wide distribution of entropy for TADs that contain sex biased genes. Lower entropy signifies better clustering of sex biased genes with other sex biased genes.


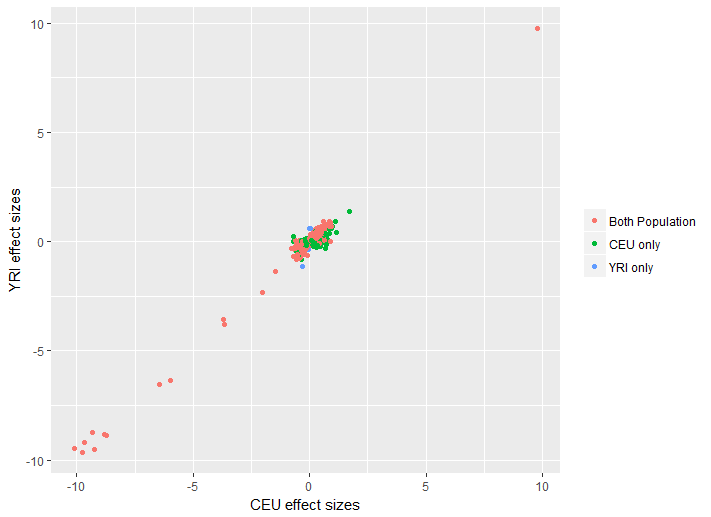


Additional file Figure 4: Effect size in log2fc of gene expression differences of the differential gene expression analyzed separately in Utah residents with Northern and Western European Ancestry (CEU) and Yoruba (YRI) populations. For clarity, only the genes that are significantly differentially expressed by sex (sDEG) are displayed. Genes that are found to be significantly differentially expressed when both CEU and YRI populations are analyzed together are plotted in orange, while genes that are found to be sDEGs when YRI samples are analyzed alone are plotted in blue, and sDEGs in CEU samples are plotted in green. There are strong correlations in effect sizes between the two populations(Pearson's correlation =0.71,pvalue < 2.1e-16), and most sDEGs (65%) are shared across the two populations.
