# Supplementary material for: Regulatory and evolutionary signatures of sex-biased genes on both the X chromosome and the autosomes
Source: Biol Sex Differ. 2017 Nov 2;8:35. doi: 10.1186/s13293-017-0156-4 (PMC5668987; doi:10.1186/s13293-017-0156-4)
Supplement: Supplementary file 2 — Linear regression of dN/dS, or the amount of selection pressure, as a function of the following variables. Estimate refers to the coefficient of the covariate, Std.Error referes to the standard error of that estimate. T value refers to the test statistic of the estimate and Pr(>|t|) refer to the P value of that covariate. XYpair refers to whether the gene is part of an XY homologous pair. XAR or XCR refers to whether the gene belongs on the X added region on the X chromosome or the X conserved region strata, escape status refers to whether the gene is classified as an escape gene, and gene expression refers to the average level of gene expression among 264 female samples. Table S2. Linear regression of dN/dS, or the amount of selection pressure, as a function of the following variables. Estimate refers to the coefficient of the covariate, Std.Error referes to the standard error of that estimate. T value refers to the test statistic of the estimate and Pr(>|t|) refer to the p value of that covariate. Average gene expression refers to average level of gene expression among 462 samples. Gene bias_female refers to whether the gene is classified as female-biased, and the coefficient refers to the change in dnds between female-biased genes to genes without sex bias. Gene bias_male refers similarly to genes classified as male-biased. Gene expression breadth refers to the number of tissues the gene is expressed in. Table S3. Sex-biased genes (sDEG) (587 genes) from LCL data and GTex data (1308) and their relationship to replication timing data in different cell lines, based on the Spearman’s Rho between replication timing values and log2fc of gene expression between females and males. Table S4(a). GSEA results for gene regions that are enriched in female biased sDEGs. Table S4(b). ToppFun results for gene regions that are enriched in male-biased sDEGs. Table S5(a). Disease gene sets enriched in sex biased genes, as found by ToppFun. Table S5(b). GO terms enriched in sex bia [file 13293_2017_156_MOESM2_ESM.docx]

Additional file Table 1: linear regression of dN/dS, or the amount of selection pressure, as a function of the following variables. Estimate refers to the coefficient of the covariate, Std.Error referes to the standard error of that estimate. T value refers to the test statistic of the estimate and Pr(>|t|) refer to the p value of that covariate. XYpair refers to whether the gene is part of an XY homologous pair. XAR or XCR refers to whether the gene belongs on the X added region on the X chromosome or the X conserved region strata, escape status refers to whether the gene is classified as an escape gene, and gene expression refers to the average level of gene expression among 264 female samples.

|  | Estimate | Std. Error | t value | Pr(>\|t\|) |
| --- | --- | --- | --- | --- |
| Intercept | 0.249 | 0.018 | 13.8 | <2e-16 |
| XYPair | -0.119 | 0.0511 | -2.329 | 0.0201 |
| XAR or XCR | 0.04151 | 0.01644 | 2.525 | 0.0117 |
| Escape status | -0.005929 | 0.017 | -0.34 | 0.7339 |

Additional file Table2: linear regression of dN/dS, or the amount of selection pressure, as a function of the following variables. Estimate refers to the coefficient of the covariate, Std.Error referes to the standard error of that estimate. T value refers to the test statistic of the estimate and Pr(>|t|) refer to the p value of that covariate. Average gene expression refers to average level of gene expression among 462 samples. Gene bias_female refers to whether the gene is classified as female-biased, and the coefficient refers to the change in dnds between female-biased genes to genes without sex bias. Gene bias_male refers similarly to genes classified as male-biased . Gene expression breadth refers to the number of tissues the gene is expressed in.

|  | Estimate | Std. Error | t value | Pr(>\|t\|) |
| --- | --- | --- | --- | --- |
| Intercept | 3.402e-01 | 3.402e-01 | 11.11 | <2e-16 |
| Average gene expression | -9.145e-08 | 1.179e-06 | -0.078 | -0.078 |
| Gene bias_female | -6.950e-03 | 7.206e-02 | -0.096 | 0.9232 |
| Gene bias_male | -4.965e-02 | 7.537e-02 | -0.659 | 0.5101 |
| Gene expression breadth | -1.279e-03 | 6.300e-04 | -2.029 | 0.0425 * |

Additional file Table 3: sex-biased genes (sDEG) (587 genes) from LCL data and GTex data (1308) and their relationship to replication timing data in different cell lines, based on the Spearman’s Rho between replication timing values and log2fc of gene expression between females and males.

| **Cell Type** | **GTeX Data, Spearman’s Rho** | ***P* value** | **LCL sDEG, Spearman’s Rho** | ***P* value** |
| --- | --- | --- | --- | --- |
| SK-N-SH | 0.1033189 | 0.00282 | 0.118 | < 2.2e-16 |
| MCF-7 | 0.07615848 | 0.02786 | 0.122 | < 2.2e-16 |
| IMR-92 | 0.06626556 | 0.05576 | 0.118 | < 2.2e-16 |
| LCL | 0.02217671 | 0.5483 | 0.111 | 0.0008 |

Additional file Table 4(a). GSEA results for gene regions that are enriched in female biased sDEGs.

| Gene Set | Size Of Gene Set | Nominal P | FDR q value |
| --- | --- | --- | --- |
| [CHR19Q13](http://www.broadinstitute.org/gsea/msigdb/cards/chr19q13) | 26 | 0 | 0 |
| [CHRXP22](http://www.broadinstitute.org/gsea/msigdb/cards/chrxp22) | 24 | 0 | 0 |
| [CHRXP11](http://www.broadinstitute.org/gsea/msigdb/cards/chrxp11) | 13 | 0 | 0.001 |
| [CHR12Q13](http://www.broadinstitute.org/gsea/msigdb/cards/chr12q13) | 5 | 0 | 0.011 |

Additional file Table 4(b). ToppFun results for gene regions that are enriched in male-biased sDEGs.

| Gene Set | Genes From Input | Size Of Gene Set | Nominal P | FDR B&H |
| --- | --- | --- | --- | --- |
| Chr4Q12 | 5 | 57 | 3.582E-3 | 3.347E-2 |
| CHRYQ11 | 3 | 12 | 5.762E-5 | 3.34E-2 |
| CHRYp11.3 | 3 | 16 | 1.439E-4 | 9.496E-3 |

Additional file Table 5(a): Disease gene sets enriched in sex biased genes, as found by ToppFun

| Name | pValue | FDR B&H | FDR B&Y | Bonferroni | Genes from Input | Genes in Annotation |
| --- | --- | --- | --- | --- | --- | --- |
| XX males | 1.15E-06 | 3.64E-03 | 3.14E-02 | 3.64E-03 | [6](https://toppgene.cchmc.org/showQueryTerms.jsp?userdata_id=3e9162c9-3aea-42b1-82cc-fbd4765d1261&feature=dis&row=0) | [17](https://toppgene.cchmc.org/showTermDetail.jsp?userdata_id=3e9162c9-3aea-42b1-82cc-fbd4765d1261&category=Disease&id=umls:C0432475) |
| Leukemogenesis | 1.17E-05 | 1.45E-02 | 1.26E-01 | 3.72E-02 | [33](https://toppgene.cchmc.org/showQueryTerms.jsp?userdata_id=3e9162c9-3aea-42b1-82cc-fbd4765d1261&feature=dis&row=1) | [660](https://toppgene.cchmc.org/showTermDetail.jsp?userdata_id=3e9162c9-3aea-42b1-82cc-fbd4765d1261&category=Disease&id=umls:C0598766) |
| B-Cell Lymphomas | 1.37E-05 | 1.45E-02 | 1.26E-01 | 4.36E-02 | [30](https://toppgene.cchmc.org/showQueryTerms.jsp?userdata_id=3e9162c9-3aea-42b1-82cc-fbd4765d1261&feature=dis&row=2) | [576](https://toppgene.cchmc.org/showTermDetail.jsp?userdata_id=3e9162c9-3aea-42b1-82cc-fbd4765d1261&category=Disease&id=umls:C0079731) |
| Chronic Obstructive Airway Disease | 4.84E-05 | 3.84E-02 | 3.32E-01 | 1.54E-01 | [26](https://toppgene.cchmc.org/showQueryTerms.jsp?userdata_id=3e9162c9-3aea-42b1-82cc-fbd4765d1261&feature=dis&row=3) | [497](https://toppgene.cchmc.org/showTermDetail.jsp?userdata_id=3e9162c9-3aea-42b1-82cc-fbd4765d1261&category=Disease&id=umls:C0024117) |

Additional file Table 5(b): GO terms enriched in sex biased genes, as found by ToppFun

| ID | | Name | | pValue | FDR B&H | FDR B&Y | Bonferroni | Genes from Input | Genes in Annotation |
| --- | --- | --- | --- | --- | --- | --- | --- | --- | --- |
| GO:0032452 | | histone demethylase activity | | 2.84E-05 | 3.47E-02 | 2.67E-01 | 3.47E-02 | [6](https://toppgene.cchmc.org/showQueryTerms.jsp?userdata_id=3e9162c9-3aea-42b1-82cc-fbd4765d1261&feature=gof&row=0) | [26](https://toppgene.cchmc.org/showTermDetail.jsp?userdata_id=3e9162c9-3aea-42b1-82cc-fbd4765d1261&category=GeneOntologyMolecularFunction&id=GO:0032452) |
| ID | Name | | pValue | | FDR B&H | FDR B&Y | Bonferroni | Genes from Input | Genes in Annotation |
| GO:0005773 | vacuole | | 1.33E-05 | | 7.64E-03 | 5.29E-02 | 7.64E-03 | [53](https://toppgene.cchmc.org/showQueryTerms.jsp?userdata_id=3e9162c9-3aea-42b1-82cc-fbd4765d1261&feature=goc&row=0) | [1223](https://toppgene.cchmc.org/showTermDetail.jsp?userdata_id=3e9162c9-3aea-42b1-82cc-fbd4765d1261&category=GeneOntologyCellularComponent&id=GO:0005773) |
| GO:0005768 | endosome | | 1.49E-04 | | 2.38E-02 | 1.65E-01 | 8.54E-02 | [37](https://toppgene.cchmc.org/showQueryTerms.jsp?userdata_id=3e9162c9-3aea-42b1-82cc-fbd4765d1261&feature=goc&row=1) | [826](https://toppgene.cchmc.org/showTermDetail.jsp?userdata_id=3e9162c9-3aea-42b1-82cc-fbd4765d1261&category=GeneOntologyCellularComponent&id=GO:0005768) |
| GO:0005924 | cell-substrate adherens junction | | 2.10E-04 | | 2.38E-02 | 1.65E-01 | 1.20E-01 | [22](https://toppgene.cchmc.org/showQueryTerms.jsp?userdata_id=3e9162c9-3aea-42b1-82cc-fbd4765d1261&feature=goc&row=2) | [398](https://toppgene.cchmc.org/showTermDetail.jsp?userdata_id=3e9162c9-3aea-42b1-82cc-fbd4765d1261&category=GeneOntologyCellularComponent&id=GO:0005924) |
| GO:0000323 | lytic vacuole | | 2.10E-04 | | 2.38E-02 | 1.65E-01 | 1.20E-01 | [27](https://toppgene.cchmc.org/showQueryTerms.jsp?userdata_id=3e9162c9-3aea-42b1-82cc-fbd4765d1261&feature=goc&row=3) | [539](https://toppgene.cchmc.org/showTermDetail.jsp?userdata_id=3e9162c9-3aea-42b1-82cc-fbd4765d1261&category=GeneOntologyCellularComponent&id=GO:0000323) |
| GO:0005764 | lysosome | | 2.10E-04 | | 2.38E-02 | 1.65E-01 | 1.20E-01 | [27](https://toppgene.cchmc.org/showQueryTerms.jsp?userdata_id=3e9162c9-3aea-42b1-82cc-fbd4765d1261&feature=goc&row=4) | [539](https://toppgene.cchmc.org/showTermDetail.jsp?userdata_id=3e9162c9-3aea-42b1-82cc-fbd4765d1261&category=GeneOntologyCellularComponent&id=GO:0005764) |

| Additional file Table 5 (c): Pubmed gene sets enriched in sex biased genes, as found by ToppFun   \| Name \| pValue \| FDR B&H \| FDR B&Y \| Bonferroni \| Genes from Input \| Genes in Annotation \| \| --- \| --- \| --- \| --- \| --- \| --- \| --- \| \| Ubiquitin-mediated proteolysis of HuR by heat shock. \| 8.49E-25 \| 2.85E-20 \| 3.14E-19 \| 2.85E-20 \| [69](https://toppgene.cchmc.org/showQueryTerms.jsp?userdata_id=3e9162c9-3aea-42b1-82cc-fbd4765d1261&feature=pm&row=0) \| [1672](https://toppgene.cchmc.org/showTermDetail.jsp?userdata_id=3e9162c9-3aea-42b1-82cc-fbd4765d1261&category=Pubmed&id=19322201) \| \| Oligo-capping: a simple method to replace the cap structure of eukaryotic mRNAs with oligoribonucleotides. \| 9.38E-19 \| 1.58E-14 \| 1.73E-13 \| 3.15E-14 \| [64](https://toppgene.cchmc.org/showQueryTerms.jsp?userdata_id=3e9162c9-3aea-42b1-82cc-fbd4765d1261&feature=pm&row=1) \| [1881](https://toppgene.cchmc.org/showTermDetail.jsp?userdata_id=3e9162c9-3aea-42b1-82cc-fbd4765d1261&category=Pubmed&id=8125298) \| \| Sex differences in structure and expression of the sex chromosome genes CHD1Z and CHD1W in zebra finches. \| 3.75E-16 \| 4.20E-12 \| 4.62E-11 \| 1.26E-11 \| [9](https://toppgene.cchmc.org/showQueryTerms.jsp?userdata_id=3e9162c9-3aea-42b1-82cc-fbd4765d1261&feature=pm&row=2) \| [13](https://toppgene.cchmc.org/showTermDetail.jsp?userdata_id=3e9162c9-3aea-42b1-82cc-fbd4765d1261&category=Pubmed&id=14660691) \| \| Proteomic analysis of podocyte exosome-enriched fraction from normal human urine. \| 1.50E-14 \| 1.26E-10 \| 1.38E-09 \| 5.03E-10 \| [54](https://toppgene.cchmc.org/showQueryTerms.jsp?userdata_id=3e9162c9-3aea-42b1-82cc-fbd4765d1261&feature=pm&row=3) \| [1716](https://toppgene.cchmc.org/showTermDetail.jsp?userdata_id=3e9162c9-3aea-42b1-82cc-fbd4765d1261&category=Pubmed&id=23376485) \| \| A Dynamic Protein Interaction Landscape of the Human Centrosome-Cilium Interface. \| 5.47E-13 \| 3.67E-09 \| 4.04E-08 \| 1.84E-08 \| [53](https://toppgene.cchmc.org/showQueryTerms.jsp?userdata_id=3e9162c9-3aea-42b1-82cc-fbd4765d1261&feature=pm&row=4) \| [1820](https://toppgene.cchmc.org/showTermDetail.jsp?userdata_id=3e9162c9-3aea-42b1-82cc-fbd4765d1261&category=Pubmed&id=26638075) \| |  |
| --- | --- | --- | --- | --- | --- | --- | --- | --- | --- | --- | --- | --- | --- | --- | --- | --- | --- | --- | --- | --- | --- | --- | --- | --- | --- | --- | --- | --- | --- | --- | --- | --- | --- | --- | --- | --- | --- | --- | --- | --- | --- | --- | --- |

Additional file Table 5 (d): pathway analysis of sex biased genes using gene sets from MSigDBC2, as found by ToppFun.

| Name | pValue | FDR B&H | FDR B&Y | Bonferroni | Genes from Input | Genes in Annotation |
| --- | --- | --- | --- | --- | --- | --- |
| Genes that escape X inactivation. | 3.48E-17 | 2.55E-13 | 2.42E-12 | 2.55E-13 | [11](https://toppgene.cchmc.org/showQueryTerms.jsp?userdata_id=3e9162c9-3aea-42b1-82cc-fbd4765d1261&feature=ct&row=0) | [13](https://toppgene.cchmc.org/showTermDetail.jsp?userdata_id=3e9162c9-3aea-42b1-82cc-fbd4765d1261&category=Coexpression&id=M16091) |
| Up-regulated genes detecting gender effects in global expression profiling studies. | 1.04E-15 | 3.82E-12 | 3.62E-11 | 7.64E-12 | [9](https://toppgene.cchmc.org/showQueryTerms.jsp?userdata_id=3e9162c9-3aea-42b1-82cc-fbd4765d1261&feature=ct&row=1) | [9](https://toppgene.cchmc.org/showTermDetail.jsp?userdata_id=3e9162c9-3aea-42b1-82cc-fbd4765d1261&category=Coexpression&id=M17072) |
| Genes up-regulated in HL-60 cells (acute promyelocytic leukemia, APL) after treatment with the aminopeptidase inhibitor tosedostat (CHR-2797) [PubChem=15547703] for 24 h. | 9.83E-09 | 2.40E-05 | 2.28E-04 | 7.21E-05 | [44](https://toppgene.cchmc.org/showQueryTerms.jsp?userdata_id=3e9162c9-3aea-42b1-82cc-fbd4765d1261&feature=ct&row=2) | [782](https://toppgene.cchmc.org/showTermDetail.jsp?userdata_id=3e9162c9-3aea-42b1-82cc-fbd4765d1261&category=Coexpression&id=M17915) |
| Down-regulated genes in head and neck cancer compared to cervical carcinoma samples. | 1.37E-07 | 2.50E-04 | 2.37E-03 | 1.00E-03 | [8](https://toppgene.cchmc.org/showQueryTerms.jsp?userdata_id=3e9162c9-3aea-42b1-82cc-fbd4765d1261&feature=ct&row=3) | [29](https://toppgene.cchmc.org/showTermDetail.jsp?userdata_id=3e9162c9-3aea-42b1-82cc-fbd4765d1261&category=Coexpression&id=M5126) |
| Genes up-regulated in HL-60 cells (acute promyelocytic leukemia, APL) after treatment with the aminopeptidase inhibitor tosedostat (CHR-2797) [PubChem=15547703] for 6 h. | 4.00E-07 | 5.21E-04 | 4.94E-03 | 2.93E-03 | [46](https://toppgene.cchmc.org/showQueryTerms.jsp?userdata_id=3e9162c9-3aea-42b1-82cc-fbd4765d1261&feature=ct&row=4) | [952](https://toppgene.cchmc.org/showTermDetail.jsp?userdata_id=3e9162c9-3aea-42b1-82cc-fbd4765d1261&category=Coexpression&id=M8144) |

Additional file Table 5 (e): Gene families enriched in sex biased genes, as found by ToppFun.

| Name | pValue | FDR B&H | FDR B&Y | Bonferroni | Genes from Input | Genes in Annotation |
| --- | --- | --- | --- | --- | --- | --- |
| Zinc fingers, C2H2-type | 4.49E-06 | 1.84E-04 | 7.92E-04 | 1.84E-04 | [21](https://toppgene.cchmc.org/showQueryTerms.jsp?userdata_id=3e9162c9-3aea-42b1-82cc-fbd4765d1261&feature=gf&row=0) | [514](https://toppgene.cchmc.org/showTermDetail.jsp?userdata_id=3e9162c9-3aea-42b1-82cc-fbd4765d1261&category=GeneFamily&id=ZNF) |
| Ubiquitin specific peptidases | 4.48E-05 | 9.17E-04 | 3.95E-03 | 1.84E-03 | [6](https://toppgene.cchmc.org/showQueryTerms.jsp?userdata_id=3e9162c9-3aea-42b1-82cc-fbd4765d1261&feature=gf&row=1) | [48](https://toppgene.cchmc.org/showTermDetail.jsp?userdata_id=3e9162c9-3aea-42b1-82cc-fbd4765d1261&category=GeneFamily&id=USP) |
| Tripartite motif-containing family | 2.19E-03 | 2.59E-02 | 1.12E-01 | 8.99E-02 | [5](https://toppgene.cchmc.org/showQueryTerms.jsp?userdata_id=3e9162c9-3aea-42b1-82cc-fbd4765d1261&feature=gf&row=2) | [67](https://toppgene.cchmc.org/showTermDetail.jsp?userdata_id=3e9162c9-3aea-42b1-82cc-fbd4765d1261&category=GeneFamily&id=TRIM) |
| RING-type zinc fingers | 2.53E-03 | 2.59E-02 | 1.12E-01 | 1.04E-01 | [9](https://toppgene.cchmc.org/showQueryTerms.jsp?userdata_id=3e9162c9-3aea-42b1-82cc-fbd4765d1261&feature=gf&row=3) | [213](https://toppgene.cchmc.org/showTermDetail.jsp?userdata_id=3e9162c9-3aea-42b1-82cc-fbd4765d1261&category=GeneFamily&id=RNF) |
| CD molecules | 4.47E-03 | 3.66E-02 | 1.58E-01 | 1.83E-01 | [10](https://toppgene.cchmc.org/showQueryTerms.jsp?userdata_id=3e9162c9-3aea-42b1-82cc-fbd4765d1261&feature=gf&row=4) | [276](https://toppgene.cchmc.org/showTermDetail.jsp?userdata_id=3e9162c9-3aea-42b1-82cc-fbd4765d1261&category=GeneFamily&id=CD) |

Additional file Table 6 (a): Domains enriched in female biased genes, as found by ToppFun.

| Name | Source | pValue | FDR B&H | FDR B&Y | Bonferroni | Genes from Input | Genes in Annotation |
| --- | --- | --- | --- | --- | --- | --- | --- |
| KRAB | [Pfam](http://pfam.sanger.ac.uk/family/PF01352) | 9.52E-06 | 3.23E-03 | 2.44E-02 | 1.01E-02 | [16](https://toppgene.cchmc.org/showQueryTerms.jsp?userdata_id=e0905c26-0875-40fa-ac27-6b5537e6fc27&feature=dt&row=0) | [334](https://toppgene.cchmc.org/showTermDetail.jsp?userdata_id=e0905c26-0875-40fa-ac27-6b5537e6fc27&category=Domain&id=PF01352) |
| KRAB | [PROSITE](http://www.expasy.org/cgi-bin/nicedoc.pl?PS50805) | 1.10E-05 | 3.23E-03 | 2.44E-02 | 1.17E-02 | [16](https://toppgene.cchmc.org/showQueryTerms.jsp?userdata_id=e0905c26-0875-40fa-ac27-6b5537e6fc27&feature=dt&row=1) | [338](https://toppgene.cchmc.org/showTermDetail.jsp?userdata_id=e0905c26-0875-40fa-ac27-6b5537e6fc27&category=Domain&id=PS50805) |
| KRAB | [SMART](http://smart.embl-heidelberg.de/smart/do_annotation.pl?BLAST=DUMMY&DOMAIN=KRAB) | 1.64E-05 | 3.23E-03 | 2.44E-02 | 1.74E-02 | [16](https://toppgene.cchmc.org/showQueryTerms.jsp?userdata_id=e0905c26-0875-40fa-ac27-6b5537e6fc27&feature=dt&row=2) | [349](https://toppgene.cchmc.org/showTermDetail.jsp?userdata_id=e0905c26-0875-40fa-ac27-6b5537e6fc27&category=Domain&id=SM00349) |
| Krueppel-associated box | [InterPro](http://www.ebi.ac.uk/interpro/IEntry?ac=IPR001909) | 1.70E-05 | 3.23E-03 | 2.44E-02 | 1.80E-02 | [16](https://toppgene.cchmc.org/showQueryTerms.jsp?userdata_id=e0905c26-0875-40fa-ac27-6b5537e6fc27&feature=dt&row=3) | [350](https://toppgene.cchmc.org/showTermDetail.jsp?userdata_id=e0905c26-0875-40fa-ac27-6b5537e6fc27&category=Domain&id=IPR001909) |
| Znf C2H2/integrase DNA-bd | [InterPro](http://www.ebi.ac.uk/interpro/IEntry?ac=IPR013087) | 2.10E-05 | 3.23E-03 | 2.44E-02 | 2.23E-02 | [23](https://toppgene.cchmc.org/showQueryTerms.jsp?userdata_id=e0905c26-0875-40fa-ac27-6b5537e6fc27&feature=dt&row=4) | [659](https://toppgene.cchmc.org/showTermDetail.jsp?userdata_id=e0905c26-0875-40fa-ac27-6b5537e6fc27&category=Domain&id=IPR013087) |

Additional file Table 6 (b): KEGG pathways enriched in female biased genes, as found by GSEA.

| **GS DETAILS** | **ES** | **NES** | **NOM p-val** | **FWER p-val** |
| --- | --- | --- | --- | --- |
|  |  |  |  |  |
|  |  |  |  |  |
| [KEGG_OOCYTE_MEIOSIS](http://www.broadinstitute.org/gsea/msigdb/cards/KEGG_OOCYTE_MEIOSIS) | 4 | 0.79 | 0.048 | 0.835 |
| [KEGG_METABOLISM_OF_XENOBIOTICS_BY_CYTOCHROME_P450](http://www.broadinstitute.org/gsea/msigdb/cards/KEGG_METABOLISM_OF_XENOBIOTICS_BY_CYTOCHROME_P450) | 3 | 0.82 | 0.055 | 0.892 |
| [KEGG_GLUTATHIONE_METABOLISM](http://www.broadinstitute.org/gsea/msigdb/cards/KEGG_GLUTATHIONE_METABOLISM) | 3 | 0.82 | 0.054 | 0.899 |
| [KEGG_DRUG_METABOLISM_CYTOCHROME_P450](http://www.broadinstitute.org/gsea/msigdb/cards/KEGG_DRUG_METABOLISM_CYTOCHROME_P450) | 3 | 0.82 | 0.05 | 0.9 |
| [KEGG_RETINOL_METABOLISM](http://www.broadinstitute.org/gsea/msigdb/cards/KEGG_RETINOL_METABOLISM) | 1 | 0.99 | 0.023 | 0.999 |
| [HELLER_SILENCED_BY_METHYLATION_UP](http://www.broadinstitute.org/gsea/msigdb/cards/HELLER_SILENCED_BY_METHYLATION_UP) | 10 | 0.88 | 0.004 | 0.528 |

Additional file Table 7: pathway analysis of sex biased genes in GSEA, using custom gene sets from (Siddani, Pochineni et al. 2013, Yang, Tang et al. 2013, Consortium 2014, Okada, Wu et al. 2014)

| Gene Set For Various Diseases | Gene set size (restricting to dataset) | Nominal *P* value | FDR q value |
| --- | --- | --- | --- |
| SLE_YANG | 19 | 0.041 | 0.16 |
| Rheumatoid Arthritis 2014 (RA_2014) | 16 | 0.127 | 0.13 |
| SCHIZOPHRENIA | 55 | 0.841 | 0.84 |

Additional file Table 8 (a): Gene families enriched in male biased genes

| Name | pValue | FDR B&H | FDR B&Y | Bonferroni | Genes from Input | Genes in Annotation |
| --- | --- | --- | --- | --- | --- | --- |
| Tripartite motif-containing family | 4.16E-05 | 1.08E-03 | 4.17E-03 | 1.08E-03 | [5](https://toppgene.cchmc.org/showQueryTerms.jsp?userdata_id=de3a137f-65f6-4d30-b2ca-34699918c81c&feature=gf&row=0) | [67](https://toppgene.cchmc.org/showTermDetail.jsp?userdata_id=de3a137f-65f6-4d30-b2ca-34699918c81c&category=GeneFamily&id=TRIM) |
| Ubiquitin specific peptidases | 1.70E-04 | 2.22E-03 | 8.54E-03 | 4.43E-03 | [4](https://toppgene.cchmc.org/showQueryTerms.jsp?userdata_id=de3a137f-65f6-4d30-b2ca-34699918c81c&feature=gf&row=1) | [48](https://toppgene.cchmc.org/showTermDetail.jsp?userdata_id=de3a137f-65f6-4d30-b2ca-34699918c81c&category=GeneFamily&id=USP) |
| Zinc fingers, FYVE type | 7.72E-04 | 6.69E-03 | 2.58E-02 | 2.01E-02 | [3](https://toppgene.cchmc.org/showQueryTerms.jsp?userdata_id=de3a137f-65f6-4d30-b2ca-34699918c81c&feature=gf&row=2) | [31](https://toppgene.cchmc.org/showTermDetail.jsp?userdata_id=de3a137f-65f6-4d30-b2ca-34699918c81c&category=GeneFamily&id=ZFYVE) |
| CD molecules | 1.03E-03 | 6.72E-03 | 2.59E-02 | 2.69E-02 | [7](https://toppgene.cchmc.org/showQueryTerms.jsp?userdata_id=de3a137f-65f6-4d30-b2ca-34699918c81c&feature=gf&row=3) | [276](https://toppgene.cchmc.org/showTermDetail.jsp?userdata_id=de3a137f-65f6-4d30-b2ca-34699918c81c&category=GeneFamily&id=CD) |
| RING-type zinc fingers | 1.44E-03 | 7.48E-03 | 2.88E-02 | 3.74E-02 | [6](https://toppgene.cchmc.org/showQueryTerms.jsp?userdata_id=de3a137f-65f6-4d30-b2ca-34699918c81c&feature=gf&row=4) | [213](https://toppgene.cchmc.org/showTermDetail.jsp?userdata_id=de3a137f-65f6-4d30-b2ca-34699918c81c&category=GeneFamily&id=RNF) |

Supplementary Table 8 (b): MSigDB gene sets enriched in male biased genes, as found by ToppFUn.

| Name | pValue | FDR B&H | FDR B&Y | Bonferroni | Genes from Input | Genes in Annotation |
| --- | --- | --- | --- | --- | --- | --- |
| Up-regulated genes detecting gender effects in global expression profiling studies. | 1.26E-18 | 7.90E-15 | 7.36E-14 | 7.90E-15 | [9](https://toppgene.cchmc.org/showQueryTerms.jsp?userdata_id=de3a137f-65f6-4d30-b2ca-34699918c81c&feature=ct&row=0) | [9](https://toppgene.cchmc.org/showTermDetail.jsp?userdata_id=de3a137f-65f6-4d30-b2ca-34699918c81c&category=Coexpression&id=M17072) |
| Down-regulated genes in head and neck cancer compared to cervical carcinoma samples. | 4.39E-10 | 1.38E-06 | 1.29E-05 | 2.76E-06 | [8](https://toppgene.cchmc.org/showQueryTerms.jsp?userdata_id=de3a137f-65f6-4d30-b2ca-34699918c81c&feature=ct&row=1) | [29](https://toppgene.cchmc.org/showTermDetail.jsp?userdata_id=de3a137f-65f6-4d30-b2ca-34699918c81c&category=Coexpression&id=M5126) |
| Genes up-regulated in B lymphocytes at 6 h after exprosure to 10 Gy dose of ionizing radiation. | 1.42E-06 | 2.98E-03 | 2.78E-02 | 8.95E-03 | [11](https://toppgene.cchmc.org/showQueryTerms.jsp?userdata_id=de3a137f-65f6-4d30-b2ca-34699918c81c&feature=ct&row=2) | [166](https://toppgene.cchmc.org/showTermDetail.jsp?userdata_id=de3a137f-65f6-4d30-b2ca-34699918c81c&category=Coexpression&id=M2588) |
| Genes forming the macrophage-enriched metabolic network (MEMN) claimed to have a causal relationship with the metabolic syndrom traits. | 9.64E-06 | 1.52E-02 | 1.41E-01 | 6.06E-02 | [30](https://toppgene.cchmc.org/showQueryTerms.jsp?userdata_id=de3a137f-65f6-4d30-b2ca-34699918c81c&feature=ct&row=3) | [1210](https://toppgene.cchmc.org/showTermDetail.jsp?userdata_id=de3a137f-65f6-4d30-b2ca-34699918c81c&category=Coexpression&id=M1920) |
| Genes up-regulated in acute myeloid leukemia (AML) with respect to cellular localization of NPM1 [GeneID=4869]: cytoplasmic vs. nucleolar. | 1.67E-05 | 1.74E-02 | 1.63E-01 | 1.05E-01 | [9](https://toppgene.cchmc.org/showQueryTerms.jsp?userdata_id=de3a137f-65f6-4d30-b2ca-34699918c81c&feature=ct&row=4) | [140](https://toppgene.cchmc.org/showTermDetail.jsp?userdata_id=de3a137f-65f6-4d30-b2ca-34699918c81c&category=Coexpression&id=M9377) |

Additional file Table 9: Pathways enriched in transcription factors enriched for female biased genes.

| Name | Source | pValue | FDR B&H | FDR B&Y | Bonferroni | Genes from Input | Genes in Annotation |
| --- | --- | --- | --- | --- | --- | --- | --- |
| Signaling mediated by p38-alpha and p38-beta | BioSystems: Pathway Interaction Database | 2.76E-04 | 9.71E-03 | 4.62E-02 | 1.80E-02 | 2 | 35 |
| TNF-alpha/NF-kB Signaling Pathway | BioSystems: WikiPathways | 2.99E-04 | 9.71E-03 | 4.62E-02 | 1.94E-02 | 3 | 196 |
| Regulation of retinoblastoma protein | BioSystems: Pathway Interaction Database | 7.10E-04 | 1.54E-02 | 7.32E-02 | 4.61E-02 | 2 | 56 |
| Wnt signaling pathway | PantherDB | 1.09E-03 | 1.77E-02 | 8.43E-02 | 7.08E-02 | 3 | 305 |
| Glucocorticoid receptor regulatory network | BioSystems: Pathway Interaction Database | 1.52E-03 | 1.97E-02 | 9.38E-02 | 9.85E-02 | 2 | 82 |
